# Examining the Relation between Caregiver Mental Health and Student Outcomes in Rural China

**DOI:** 10.3390/ijerph182312613

**Published:** 2021-11-30

**Authors:** Huan Wang, Claire Cousineau, Yuwei Adeline Hu, Grace Hu, Sunny Qi, Adrian Sun, Helen Wu, Scott Rozelle, Manpreet Singh

**Affiliations:** 1 Stanford Center on China’s Economy and Institutions, Stanford University, Palo Alto, CA 94305-6055, USA; huanw@stanford.edu (H.W.); clairece@stanford.edu (C.C.); adelinehu22@gmail.com (Y.A.H.); gracehu4320@gmail.com (G.H.); sunny02px2022@gmail.com (S.Q.); aysun@exeter.edu (A.S.); helenwu3435@sina.com (H.W.); rozelle@stanford.edu (S.R.); 2 Department of Psychiatry and Behavioral Sciences, Division of Child and Adolescent Psychiatry and Child Development, School of Medicine, Stanford University, Palo Alto, CA 94305-5719, USA

**Keywords:** caregiver’s mental health, student outcomes, rural China

## Abstract

Research continues to highlight the central relationship between caregivers’ mental health and their children’s development. This study examined the relation between primary caregivers’ mental health and school-aged children’s outcomes, including student mental health, resilience, and academic performance, in rural China. Using cross-sectional data from economically poor areas in the Gansu province, 2989 students (mean age = 11.51, 53.33% male, 46.67% female) and their primary caregivers (74.2% female) completed the 21-item, self-report Depression Anxiety Stress Scale. Students also completed the 25-item Connor-Davidson Resilience Scale and a standardized math test. The results indicated a high prevalence of caregiver depression (31%), stress (39%), and anxiety (24%). Characteristics that were significantly correlated with caregiver mental health issues included being a grandparent, having a low socioeconomic status and low education level, and living in a household with at least one migrant worker. Apart from caregiver stress and student resilience, caregiver mental health issues were negatively correlated with all student outcomes, including student mental health, resilience, and academic performance. Although additional empirical research is needed to investigate the associations between caregiver mental health and student outcomes, our results suggest that rural communities could benefit greatly from programs focused on improving the mental health of caregivers and this, in turn, may have a positive impact on student outcomes.

## 1. Introduction

A growing body of research highlights a central relation between primary caregivers’ mental health and their children’s early childhood development outcomes. Specifically, prior studies have found that poor mental health, including depression and anxiety, among caregivers may be associated with poor health outcomes among their children [1,2,3,4]. Notably, multiple studies have found associations between maternal depressive symptoms and impaired overall health, growth, and development of offspring [5]. 

Parental depression may also negatively affect the quality of parent–child interactions, including certain aspects of a child’s mental health and well-being [6,7,8]. 

The extant literature suggests that, in addition to the impact on early childhood development, the mental health of a caregiver also may have an impact on student outcomes, including mental health, resilience, and academic performance. Specifically, compared to the offspring of parents without depression, the adolescent offspring of parents with depression have a greater risk of being victimized by both teachers and students at school, which could affect student mental health, educational attainment, and peer interactions [9]. Other research has found that the prevalence of mental health conditions, including depression and anxiety, among youth increases as parental mental health worsens [10]. Notably, depressive symptoms in adolescents have been negatively correlated with the ability to graduate from high school and, in turn, the ability to find work, a benchmark for successful transition into independence or adulthood [11,12,13]. Further, resilience can enable students to achieve academically despite unfavorable social circumstances [14]. 

A growing body of literature has examined several possible mechanisms through which caregivers can impact student outcomes. Specifically, high parental expectations and parental involvement have been consistently associated with higher levels of student achievement [15,16,17] and have been found to foster resilience [18]. Similarly, according to the literature, among the most important determinants of students’ level of resilience is the presence of a supportive and meaningful relationship with the adults in their lives [19]. Research indicates, however, that parents who experience anxiety may be more withdrawn or disengaged in parenting [14], which has the potential to negatively affect their involvement and expectations and, in turn, their children’s academic outcomes. 

Given the importance of caregiver mental health, high rates of caregiver mental health issues, such as depression, anxiety, and stress, in developing countries are of concern. Studies show that, among maternal caregivers in low- to middle-income countries (LMICs), where 90% of the world’s children live [5], the prevalence of depression, anxiety, and stress is as high as 57%, 20.7%, and 37%, respectively, although this varies depending on how the problems are identified and diagnosed [20,21,22,23]. Researchers have found several risk factors that contribute to the high prevalence of poor mental health in low-income settings, such as poverty-related and economic-adversity indicators, including low education levels, low income, and poor housing conditions [24,25,26,27,28]. Additional demographic characteristics have been identified as risk factors for poor caregiver mental health in LMICs, including child gender, caregiver age, caregiver gender, and the number of children for whom caregivers are responsible for raising. Specifically, in cultures where there is a strong male bias, the birth of a female child could contribute to caregiver mental health issues [5]. Similarly, female, middle-aged caregivers have been found to be at higher risk for depression as a result of the adverse circumstances experienced by many women in developing countries [24,27]. Finally, mothers who perceive that they have too many children or mothers who have more than five children also are at a heightened risk, likely due to a lack of resources and insufficient support [24,27].

Examining the relation between caregiver mental health and student outcomes is particularly important in developing settings, such as rural China, where research has documented a high prevalence of caregiver depression [23,29]. Although a study conducted in rural China found that primary caregivers experience high levels of anxiety, depression, and stress, 42%, 32%, and 30%, respectively [29], limited attention has been given to these issues beyond early childhood. Given that 75% of China’s children are educated in rural settings, the high rate of mental health issues among rural caregivers could negatively affect academic outcomes later in childhood [30]. Assessing the factors that contribute to educational outcomes is essential for rural China, where the development of a greater level of human capital is critical to economic growth and the transition to a skills-based economy [11,12,13,31,32]. 

Despite the high rate of depression among caregivers in rural China, research has focused primarily on early childhood development outcomes and has not evaluated the direct relation between caregiver mental health and student outcomes. Past studies conducted in rural China have found that mental health issues, including depression, anxiety, and stress, among caregivers are often associated with poor developmental outcomes in their children’s infancy and early childhood [23]. In this regard, a study in rural China found that children aged 0–3 years who have caregivers with depressive symptoms exhibited lower social-emotional development scores [33]. Although these studies focused on rural China, they mainly examined the development of young children and toddlers, rather than providing an understanding of the impact of caregiver mental health on older school-aged youth. As a result, there has been little research on the effects of caregiver mental health on student outcomes, including student mental health, resilience, and academic performance. Further, extant studies have focused primarily on maternal caregivers; as such, even though many primary caregivers in rural China are fathers and grandparents, few studies have focused on their roles as caregivers. 

This study seeks to address the gaps in the literature by examining the relation between caregiver mental health and outcomes of school-aged children in rural China. To do so, we formulated three objectives. First, we used standardized and internationally validated scales to document the prevalence of caregiver mental health issues in rural China, focusing on levels of self-reported depression, anxiety, and stress. Second, we measured potential factors associated with these caregiver mental health issues. Third, we examined the correlation between caregiver mental health issues and student outcomes, specifically regarding student mental health, resilience, and academic performance, by exploring potential underlying mechanisms. Although the term primary caregiver can encompass a variety of individuals, throughout this paper, the term is used to refer to a child’s parents or grandparents who are primarily responsible for raising the child. 

To achieve these objectives, we tested the following hypotheses. First, using the 21-item self-report Depression Anxiety Stress Scale (DASS) we tested the hypothesis that caregivers in rural China are at risk for developing mental health issues, including depression, anxiety, and stress. Second, we tested the hypothesis that there are specific student and household characteristics associated with the mental health of caregivers, including caregiver socioeconomic status, caregiver gender, whether the student is an only child, and student gender. Third, we tested the hypothesis that caregiver mental health issues are negatively associated with a range of student outcomes, including student mental health (as measured by the DASS), student resilience levels (as measured by the 25-item Connor-Davidson Resilience Scale [CD-RISC]), and student academic performance (as measured by standardized math tests). Finally, we examined the hypothesis that low parental expectations for a child and parental disengagement in a child’s academic life are mechanisms associated with lower student outcomes. 

## 2. Materials and Methods

### 2.1. Ethics Approval

Ethics approval for this study was granted by the Stanford University Institutional Review Board. Written consent forms were sent to parents or guardians of eligible students prior to inclusion in the survey.

### 2.2. Study Location and Sampling

The data presented in this study were collected in October 2020 from 30 rural schools located in northwest China’s Gansu province. The per capita yearly income of households in the sample area was approximately 4234 USD, which is roughly equivalent to the national average income of Chinese residents (4033 USD) [34]. Our sample area is, thus, representative of poor rural areas in China, where nearly half of the nation’s population resides [35]. Moreover, 58% of the population in the sample area were rural residents [34]. 

To select our study sample, we obtained a list of all schools from the local education bureau and randomly chose 30 (20 primary schools and 10 junior high schools) for inclusion in our sample. We conducted our studies among fourth and fifth graders in each of the sample primary schools, as well as seventh and eighth graders in each of the sample junior high schools. There were three main reasons for sampling students from these grades. First, students in fourth grade or above have the necessary literary and numeracy skills to complete survey questionnaires; our pilot survey indicated that students in younger grades had trouble understanding and completing questions regarding demographic information. Second, to obtain an accurate assessment of student learning, academic achievement tests are usually given to students who have more than four years of formal schooling at the time of testing. This is a common practice in educational studies [36,37]. Third, students in China’s graduating grades (sixth grade in primary school and ninth grade in junior high school) may face greater academic pressure and spend more time on homework and remedial academic work than their younger peers under China’s competitive education system [38]. As such, mental health issues may affect students in these grades differently, as they face increasing academic pressure. 

Due to financial constraints, we randomly selected two classes at most from the selected grades of each school. Specifically, if there were only one or two classes in a grade, all classes in this grade were selected. If there were more than two classes in a grade, we randomly selected two classes. All students in each sample class who were present on the day of the survey were selected to participate in the survey. All students were asked to take home a form for their primary caregiver to complete. We distributed 3148 caregiver survey forms and received 2989 completed forms, giving a response rate of over 95%. We checked the differences between the attrition sample and the remaining sample and found that there were no statistically significant differences between the two samples on a variety of student and caregiver covariates, such as student age, whether the student was the only child, student afterschool activities, parental education level, and family wealth level. In total, we surveyed 2989 students and their caregivers across 30 sample schools. 

Because some students’ parents migrate to China’s urban areas for work, they were not present to serve as the primary caregiver or to complete the survey form. The grandparents who lived with these parents’ children and served as their primary caregivers completed the survey form and reported their mental health status. The completed forms then were returned to the homeroom teacher and mailed to the research team.

### 2.3. Outcome Measures 

Student and caregiver mental health was measured by the 21-item self-report DASS. The DASS is a well-recognized self-rating scale of the common mental health conditions of depression, anxiety, and stress. It has been used by different age groups and validated in many contexts around the world, including in rural China. The DASS has been shown to have high reliability among Chinese students (estimated Cronbach’s alpha is greater than 0.90) [39]. In addition, our results indicate that the DASS-21 scale had good reliability among our sample participants, with Cronbach’s alpha coefficients of 0.926 for caregivers and 0.915 for students. For each of the 21 items, respondents indicated how well a statement applies to them over the past week, using a 4-point Likert scale (0 = did not apply to me at all, 1 = applied to me to some degree, 2 = applied to me to a considerable degree, and 3 = applied to me very much, or most of the time.) Each of the three DASS scales contains seven items, divided into subscales with similar content. A total score for each of the three scales was obtained by adding up the seven items for depression, anxiety, or stress and multiplying by 2 to calculate the final score; a higher score indicated a higher level of symptom severity. Each scale then was divided into “normal,” “mild,” “moderate,” “severe,” and “extremely severe,” according to the recommended cutoff scores for conventional severity labels [40,41,42]. The validity of the scale and cutoff scores have been verified in China by Wang et al. [43]. The term mental health issues is henceforth be used to describe respondents exhibiting collective symptoms, including mild, moderate, severe, and extremely severe levels of depression, anxiety, and stress, unless otherwise specified.

Student resilience was measured by the 25-item CD-RISC. The CD-RISC is a well-recognized self-rating scale of resilience that can be used by different age groups and has been validated in many contexts around the world [44], including among adolescents in China (Cronbach’s alpha coefficient of 0.89) [45]. The Cronbach’s alpha coefficient of CD-RISC for our sample students was 0.873. For each of the 25 items, respondents indicated how well a statement applies to them over the past month, using a 5-point Likert scale (0 = not true at all, 1 = rarely true, 2 = sometimes true, 3 = often true, and 4 = true nearly all the time). A total score, ranging from 0 to 100, was obtained by adding all 25 items, with higher scores indicating higher levels of resilience. 

Student academic performance was measured by a 30-min standardized math test. We utilized math as the measurement of academic performance, because achievement in math is more explicitly tied to learning experiences at school as opposed to learning experiences at home (such as reading or language achievement). To make the math test results comparable across different sample schools, we conducted our own standardized mathematics test for each sample grade in our survey. The test items for each sample grade were carefully designed with assistance from educators who were working at the local education bureau to ensure compliance with the national curriculum, and they had been used by the research team in several previous surveys to examine student academic performance in other parts of rural China. We pre-tested the exam multiple times to ensure its relevance and that time limits were appropriate. When we administered the exam in the sample schools, it was timed carefully and closely proctored by trained enumerators. All test scores were then normalized according to the distribution of scores in each grade.

### 2.4. Covariates

Covariates included basic demographic characteristics, such as student gender (male or female) and age. Information on whether students were boarding at school (yes or no) or were the only child (yes or no) was also collected. To measure the education level of each student’s parents, we asked each student to report the highest education level for both parents by choosing from five categories: illiterate, primary school (six years of education), junior high school (nine years of education), high school (12 years of education), and college and above. We then used high school attainment or above as a cutoff to create a dummy variable (>12 years education, yes or no). “Left-behind children” were classified as those students whose parents had both migrated for work for more than six months in the past year. To measure socioeconomic status, the questionnaire also asked whether the household owned seven selected items included in the National Household Income and Expenditure Survey to create a family asset index, which we categorized into quartiles. To measure student time allocation, students were asked to report the time spent on extracurricular reading after school and screen usage (minutes per day).

### 2.5. Statistical Analysis 

Our statistical analysis comprised three parts. First, we described the summary statistics of all variables for the full sample as well as the prevalence of depression, anxiety, and stress among the caregivers in our sample. Second, we conducted *t*-tests to examine the differences in the means for symptomatology between the caregiver and student demographic variables. Using multivariate logistic regression models, we estimated the associations between demographic characteristics and caregiver mental health. The outcome variable (depression, anxiety, and stress) equaled 0 if the caregiver’s DASS score was “normal” according to the conventional recommended cutoff scores. The outcome equaled 1 if the caregiver exhibited any level of mental health symptoms, including mild, moderate, severe, or extremely severe levels of symptoms. It should be noted this general approach has been used by others in the literature [23,29,33]. Finally, an ordinary least squares (OLS) linear regression model was used to conduct a multivariate analysis of the correlation between caregiver mental health and student outcomes, including student mental health, resilience, and academic performance. The following variables were included in our multivariate analysis as potential confounders: student age and gender, whether the student boarded at school, whether the student was an only child, whether extracurricular reading time after school exceeded 30 min, whether screen time exceeded 1 h per day, parental educational level, whether both parents had out-migrated for more than six months in the past year (i.e., left-behind child status), and household asset index. To account for the nested nature of the data, we clustered all standard errors at the school level.

All analyses were performed with Stata 16.1 (StataCorp LP, College Station, TX, USA). *p*-values below 0.05 were considered statistically significant.

## 3. Results

Table 1 displays the summary statistics of the caregivers and students in our sample. Over half of the sample caregivers (58.4%) were mothers. Caregivers who were fathers comprised 17.3% of the sample, while grandmothers comprised 15.8% and grandfathers comprised 8.4%. On average, the fathers in our study were 41 years old and the mothers were 38 years old. Only 24% of the fathers and 14% of the mothers had completed a high school education. Over three-quarters of the sampled families had had at least one member who had migrated for work. The mean age of sampled students was 11.5 years. Among the students in the sample, 15% had attended a boarding school and lived in a dormitory at school. In addition, 20% of the sampled students were left-behind children. The average recreational reading time of these students was 27.58 min and just over half (54%) of the sample students read for over 30 min a day as an extracurricular activity. In addition, the mean screen time of the sampled students was 21.50 min, while 12% had a screen time that exceeded one hour per day.

Table 2 presents the prevalence of depression, anxiety, and stress among the caregivers in our sample. Overall, a significant portion of our sample of primary caregivers had experienced symptoms of depression, stress, or anxiety. Within the full sample, the rates at which caregivers experienced any level of depression, anxiety, and stress were 31%, 39%, and 24%, respectively. When examining the severity of depression among our sample of caregivers, we found that 10.24% had experienced mild depressive symptoms; 14.72% had experienced moderate depressive symptoms; 4.55% had experienced severe depressive symptoms; and 1.77% had experienced extremely severe depressive symptoms. In regard to the severity of anxiety among caregivers, we found that 7.56% of our sample exhibited mild anxiety symptoms; 15.76% exhibited moderate anxiety symptoms; 7.06% exhibited severe anxiety symptoms; and 8.8% exhibited extremely severe symptoms. For the severity of stress among caregivers, we found that 9.6% showed mild stress symptoms; 9.97% showed moderate stress symptoms; 3.01% showed severe stress symptoms; and 0.94% showed extremely severe stress symptoms.

**Figure A1:**
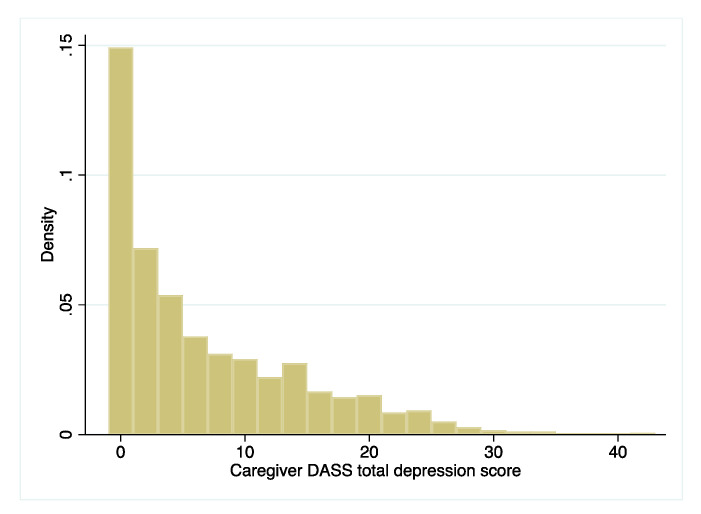
Distribution of caregiver DASS total depression scores.

**Figure A2:**
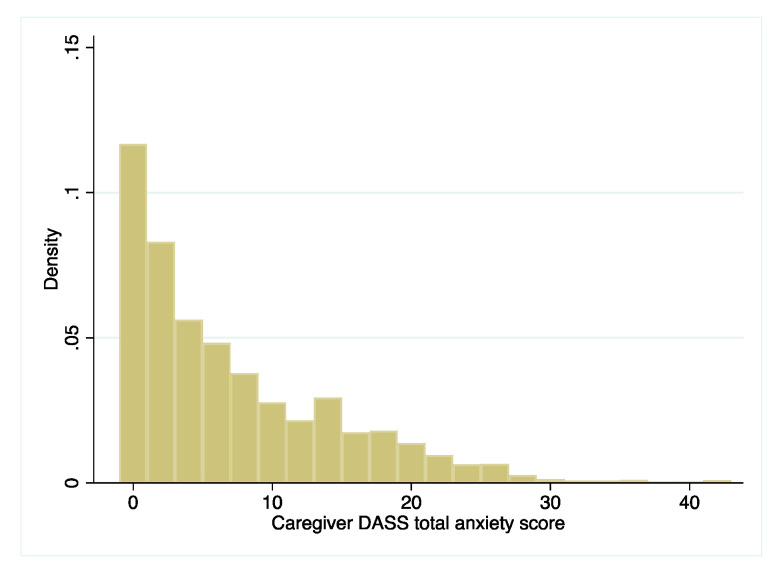
Distribution of caregiver DASS total anxiety scores.

**Figure A3:**
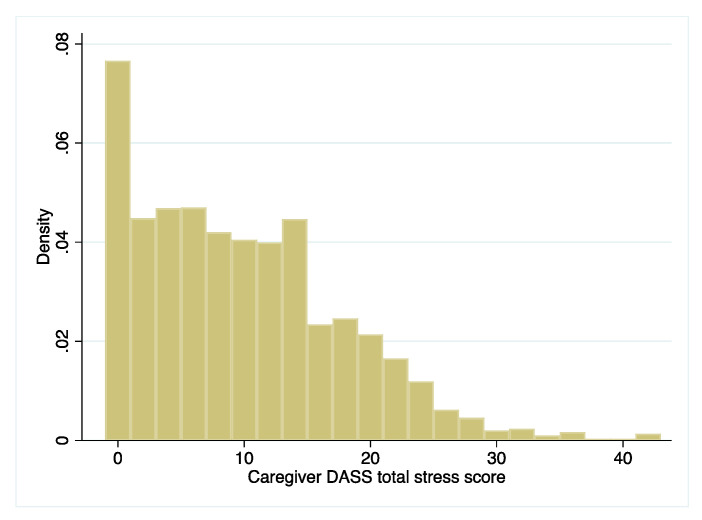
Distribution of caregiver DASS total stress scores.

As has been established, our results indicate that the DASS-21 scale has good reliability among caregivers in rural China, with a Cronbach’s alpha reliability coefficient of 0.926. The distribution of each DASS scale (depression, anxiety, and stress) total score was skewed to the left (Appendix A, see Figure A1, Figure A2 and Figure A3).

Table 3 presents the results for the differences in the prevalence of mental health issues, including depression, anxiety, and stress, among caregivers in different demographic characteristic subgroups. The results show that grandparents acting as the primary caregiver had significantly higher rates of mental health issues than parents acting as the primary caregiver. For example, 39.3% of the grandparents in our sample exhibited symptoms of depression, which was 10.5 percentage points higher than for parents acting as the primary caregiver (*p* < 0.001). In addition, compared with those who had less education, caregivers who had more than nine years of education exhibited significantly lower levels of depression (13.7 percentage points lower), anxiety (13.4 percentage points lower), and stress (10.5 percentage points lower). Finally, the results show that having family members who had out-migrated or resided in a poorer household (family asset index at bottom quartile in our sample) was linked with higher rates of caregiver depression, anxiety, and stress. For instance, our data indicate that 32.9% of caregivers living in households with at least one migrant worker exhibited depressive symptoms, which was 7.1 percentage points higher than the value for caregivers living in households without migrant workers. Similarly, 36% of caregivers with a low family asset index exhibited depressive symptoms, which was 6.5 percentage points higher than the value for caregivers who did not have a low family asset index. When comparing mental health issues by caregiver demographic characteristics, the gender of caregiver and student and whether the student had siblings were not significantly associated with an increased risk of mental health issues among caregivers. 

Table 4 displays the results of the multivariate logistic regression analysis of the correlations between student and family characteristics and the risk of mental health issues among sample caregivers. We found that certain family characteristics were significantly correlated with caregiver mental health issues. Specifically, grandparents who were the primary caregiver exhibited higher levels of depression (*β* = 0.40, *p* < 0.01), anxiety (*β* = 0.46, *p* < 0.01), and stress (*β* = 0.27, *p* < 0.05). Being a female caregiver was significantly associated with lower levels of depression (*β* = −0.26, *p* < 0.01) and stress (*β* = −0.25, *p* < 0.05). Caregivers with low educational attainment levels, specifically those who had less than a high school education, showed significantly higher levels of depression (*β* = −0.71, *p* < 0.01), anxiety (*β* = −0.56, *p* < 0.01), and stress (*β* = −0.65, *p* < 0.01). Having a family member who out-migrated for work was significantly correlated with a higher risk of caregiver anxiety (*β* = 0.20, *p* < 0.05), but not depression and stress. Finally, low family health was significantly associated with an increased risk of depression (*β* = 0.22, *p* < 0.05), anxiety (*β* = 0.26, *p* < 0.01), and stress (*β* = 0.22, *p* < 0.05) among caregivers. We found no significant correlations between mental health issues in caregivers and certain student characteristics, including student gender or whether the student was an only child. 

Table 5 shows the results of a multivariate OLS linear regression model that was used to examine the relation between caregiver mental health status and student outcomes. After controlling for student and family characteristics, the results indicated that children of caregivers who exhibited symptoms of depression, anxiety, or stress had significantly worse mental health outcomes themselves, by 24, 32, and 16 percentage points, respectively (*p* < 0.01). The results also show that student resilience levels are significantly correlated with caregiver depression (*β* = −1.39, *p* < 0.10) and anxiety (*β* = −1.85, *p* < 0.05), but not stress. Finally, significant negative associations between student academic performance and caregiver mental health issues were identified. Specifically, a student scored lower in academic performance if the caregiver exhibited depression (*β* = −0.23 SD, *p* < 0.001), anxiety (*β* = −0.21 SD, *p* < 0.001), or stress (*β* = −0.13 SD, *p* < 0.001). 

Table 6 presents the results related to potential mechanisms responsible for the effect of caregiver mental health on student outcomes. Compared to caregivers who did not experience mental health issues, caregivers who experienced depression, anxiety, or stress were less likely to expect their children to go to college by 11, 9, and 10 percentage points, respectively, and were less likely to expect their children to go to school after junior high by 5, 5, and 3 percentage points, respectively. Students were less likely to reach out to their parents for support or help if their caregiver experienced depression, anxiety, or stress by 4 percentage points across all conditions. These associations were statistically significant, even after controlling for student and family characteristic covariates.

## 4. Discussion

This paper is one of the first to examine the levels and correlates of caregiver mental health issues as well as the relation between caregiver mental health issues and student outcomes of school-aged children in rural China. Using data from 2989 students and their primary caregivers across 30 schools in poor rural areas of Gansu province, the study found that the prevalence of caregiver depression, anxiety, and stress was 31%, 39%, and 24%, respectively. Characteristics that were significantly correlated with caregiver mental health issues included being a grandparent, having a low socioeconomic status and low education level, and living in a household with at least one migrant worker. Our results also confirmed the relation between caregiver mental health issues and lower student outcomes, inclusive of student mental health, resilience, and academic performance. The presence of caregiver depression, anxiety, and stress symptoms was negatively correlated with student mental health (student mental health was 24, 32, and 16 percentage points lower, respectively). Caregiver depression and anxiety, but not stress, were correlated with student resilience levels (student resilience levels were −1.39 and −1.85 points lower, respectively). In addition, students of caregivers who exhibited symptoms of depression, anxiety, and stress scored 0.23, 0.21, and 0.13 standard deviations lower, respectively, on the standardized math exam.

Our findings in regard to the high prevalence of mental health issues among the caregivers suggest that caregiver mental health is of great concern in rural China. Overall, the portions of caregivers who exhibited symptoms of depression, anxiety, and stress were 31%, 39%, and 24%, respectively. The distribution of each DASS scale (depression, anxiety, and stress) total score was skewed to the left (Appendix A, see Figure A1, Figure A2 and Figure A3), which could be a result of the relative stigma associated with mental health disorders in China [46]. In addition, the rate of mental health issues in our sample was similar or higher than the average rates found in other studies. For example, a study that used the same DASS-21 scale found that the prevalence of depression among caregivers of young toddlers in the Qinling Mountain region of rural China was 23% [33]. Another study in a southwestern province of China also found consistent rates of depression, anxiety, and stress among rural caregivers [29]. 

We also sought to measure the factors associated with caregiver mental health issues. Consistent with the literature, our data indicate that grandparents experience higher rates of mental health issues compared to other caregivers; extant studies indicate that older, less-educated, and lower-income individuals are more susceptible to mental health disorders [33,47]. Similarly, caregivers with lower education levels and lower socioeconomic status exhibited high rates of depression, anxiety, and stress. Studies have found that having a low SES is associated with an increased risk of at least one mental health problem [33,48,49,50]. Certain factors, such as hunger and a decreased ability to obtain health care for health problems, have been found to increase the risk of caregivers developing mental health issues [51]. Caregivers of households with at least one migrant worker were also found to have higher rates of depression, anxiety, and stress in our sample. Extant research indicates that migrant workers are more likely to experience mental health disorders [52] and that parental migration negatively affects the mental health outcomes of their children [53]. 

Our findings also show, however, several factors associated with caregiver mental health issues that are inconsistent with those established in prior studies. For example, our results demonstrate that male caregivers have a higher prevalence of mental health issues when compared to female caregivers. Studies have found that, in a typical Chinese household, the income of fathers is much higher than that of mothers [54]. As such, it could be the case that families with a male primary caregiver suffer from financial or mental stress due to a decrease in income. Our data also suggest that the number of children in a household, as well as gender of the child, are not significantly correlated with caregiver mental health issues. These results could be explained by the recent abolition of China’s one-child policy. In the past, the one-child policy limited families’ reproductive freedoms and created a gender imbalance in China’s population due to a preference for male children [33]. With the abolition of the one-child policy and increasing reproductive freedom, rural families may be more inclined to enjoy a larger family rather than to experience stress over the gender of their child. 

Finally, our study is the first of its kind to use standardized tests to confirm that caregiver mental health is significantly correlated with student outcomes, including student mental health issues, resilience, and academic performance. There are several possible explanations for this. Previous studies have found that depression and anxiety in caregivers are associated with students’ internalizing problems, lower mental development, academic difficulties, and socializing issues [5,33,50,55]. Further, parents who exhibit symptoms of depression or anxiety tend to be more withdrawn and disengaged in tasks that involve their children [55]. As such, it is possible that parental disengagement could negatively affect the child’s mental health, which could subsequently negatively affect the child’s academic performance [6,7,8]. Our data also suggest that caregiver mental health is negatively correlated with student resilience. The extant literature indicates that stronger caregiver-student relationships and quality communication develops student resiliency; as has been established, decreased engagement and communication with the student could have the opposite effect [55,56]. Notably, although the symptoms of depression or anxiety of caregivers were found to be negatively correlated with student resilience in our sample, there was no significant correlation between student resilience and the stress level of caregivers. Previous research suggests that some degree of stress is necessary for students to establish resilience, which could explain the lack of association between stress and resilience in our sample [57,58,59]. 

After establishing the association between caregiver mental health and student outcomes, we examined possible mechanisms through which the two are related. As noted, previous studies have indicated the importance of parental expectations and involvement in student outcomes, including student academic performance, mental health, and resilience [15,16,17,18,19]. Our results indicate that caregivers with mental health issues are less likely to expect students to go to college or to continue schooling after junior high. Because students often rely on caregivers to guide their academic performance, they could exhibit a lack of motivation to seek higher education when their caregivers have low expectations for them [60]. Similarly, students were found to be less likely to seek help from their family if their caregiver exhibited symptoms of depression or anxiety. As extant research has established, caregivers who experience depression and anxiety have the tendency to withdraw, and students often internalize their problems when they do not feel that guidance is available [57]. As such, caregiver disengagement as a result of mental health issues could serve as a possible explanation for the relation between caregiver mental health issues and lower student outcomes.

The limitations in our study include the use of cross-sectional data, which prevented the researchers from drawing causal conclusions about the relation between caregiver mental health and student outcomes. In addition, this study relied on self-report questionnaires by students and caregivers, which created the potential for self-report biases. Finally, this study included data from only one province in China, which could decrease the generalizability of our conclusions, as the sample may not be representative of all rural China. Despite these limitations, our study makes two crucial contributions to the literature. Most importantly, this is one of a few existing studies to use standardized tests to examine the correlation between caregiver mental health and student outcomes as well as being the first of its kind in rural China. Second, this is one of the first studies conducted in rural China to investigate the mental health of caregivers beyond maternal caregivers and to include paternal and grandparent caregivers.

## 5. Conclusions

The high prevalence of mental health issues (depression, anxiety, and stress) among caregivers of rural students is an important social and health problem in rural China. Although more research is necessary to examine the causal relation between caregiver mental health and student outcomes, our study provides empirical evidence, using a large sample size, that there are significant associations between the mental health of caregivers and student outcomes, including student mental health, resilience, and academic performance. 

Our results suggest that rural communities could benefit greatly from educational programs that concern mental health among caregivers and its influence on student outcomes. Because one of the most comprehensive and well-respected institutions in rural China is its school system, we suggest that policymakers utilize the school system to reach caregivers. In doing so, policymakers can engage caregivers in programs that address the mental health of caregivers and raise awareness of the relation between caregiver mental health and student outcomes. In addition, we recommend that local hospitals establish formal relationships with schools to make their services readily available for both prevention and treatment. Perhaps most importantly, China’s top leaders need to emphasize that making China healthier in all dimensions—including mental health—is a national priority and that, as such, individuals who choose to receive treatment or reach out for help should not be stigmatized. In this regard, social media, such as WeChat, could be useful in spreading information and raising awareness about the adverse effects of caregiver mental health issues on student outcomes. We hope that our study provides insight to guide policymakers in targeting some of the most vulnerable and important populations in rural China to help shape the future of China’s children.

## Figures and Tables

**Table 1:** Summary statistics of the sample.

Variable	Frequency (*n*)	Percentage (%)	Mean (SD)	Min.	Max.
Caregiver characteristics					
Primary caregiver					
Mother	1746	58.4			
Father	518	17.3			
Grandmother	473	15.8			
Grandfather	252	8.4			
Caregiver education level					
Illiterate	255	8.5			
Primary school	1037	34.7			
Junior high school	1221	40.9			
High school	283	9.5			
College	193	6.5			
Number of migrant workers					
0	694	23.22			
1	1519	50.82			
2	671	22.45			
3	105	3.51			
Father has >12 years education (1 = yes)	703	23.5			
Mother has >12 years education (1 = yes)	429	14.4			
Father’s age (years)			41.06 (6.13)	23	71
Mother’s age (years)			38.15 (5.69)	21	66
Family asset index			0.00 (1.24)	−2.24	2.91
Student characteristics					
Student age (years)			11.51 (1.63)	7.92	15.58
Student is female	1395	46.6			
Boarding at school (1 = yes)	448	15.0			
Only child	422	14.1			
Left-behind child (1 = yes; both parents out-migrate)	607	20.1			
Standardized math test score			0.00 (0.96)	−4.02	1.9
Extracurricular reading time per day (mins)			27.58 (24.58)	0	160
Extracurricular reading time > 30 min/day (1 = yes)	1673	55.3			
Screen time per day, mins			21.50 (32.66)	0	650
Screen time exceeds 1 h/day (1 = yes)	344	11.3			
From whom student seeks help when they encounter a problem			0.87 (0.33)		
Nobody	333	11.15			
Family members	1024	34.28			
Teachers	254	8.50			
Friends	1376	46.07			

**Table 2:** Prevalence of caregiver mental health issues.

	Depression		Anxiety		Stress	
Type	Frequency (*n*)	Percentage (%)	Frequency (*n*)	Percentage (%)	Frequency (*n*)	Percentage (%)
Total abnormal	935	31.28	1171	39.18	703	23.52
Mild	306	10.24	226	7.56	287	9.6
Moderate	440	14.72	471	15.76	298	9.97
Severe	136	4.55	211	7.06	90	3.01
Extremely severe	53	1.77	263	8.8	28	0.94

**Table 3:** Distribution of caregivers’ mental health by demographic characteristics.

		Depression			Anxiety			Stress		
Characteristic	*n*	Percentage	Difference	*p*-Value	Percentage	Difference	*p*-Value	Percentage	Difference	*p*-Value
Primary caregiver										
Parents	2292	28.8	−10.5	<0.001	36.3	−12.3	<0.001	22.9	−6.0	<0.001
Grandparents	697	39.3			48.6			28.6		
Caregiver gender										
Male	770	34.4	4.6	0.018	41.3	3.2	0.116	26.0	3.5	0.047
Female	2156	29.8			38.1			22.4		
Caregiver has >9 years of education										
Yes	476	19.7	−13.7	<0.001	27.9	−13.4	<0.001	14.7	−10.5	<0.001
No	2513	33.5			41.3			25.2		
Household has at least one migrant worker										
Yes	2295	32.9	7.1	<0.001	41.3	9.2	<0.001	24.8	5.5	0.003
No	694	25.8			32.1			19.3		
Family asset index at bottom 25%										
Yes	814	36.0	6.5	0.001	45.0	8.0	<0.001	27.5	5.5	0.002
No	2175	29.5			37.0			22.0		
Student gender										
Male	1594	32.1	1.7	0.328	39.1	−0.2	0.911	23.2	0.7	0.672
Female	1395	30.4			39.3			23.9		
Student is only child										
Yes	422	32.9	1.9	0.428	39.1	−0.1	0.972	24.4	0.1	0.643
No	2,567	31.0			39.2			23.4		

**Table 4:** Logit multivariable regression of factors related to caregiver mental health.

Variable	DASS Depression	DASS Anxiety	DASS Stress
Primary caregivers are grandparents (1 = yes)	0.40 ***	0.46 ***	0.27 **
	(0.10)	(0.10)	(0.11)
Female caregiver (1 = yes)	−0.26 ***	−0.17 *	−0.25 **
	(0.09)	(0.09)	(0.10)
Caregiver has high school or above education (1 = yes)	−0.71 ***	−0.56 ***	−0.65 ***
	(0.13)	(0.12)	(0.15)
At least one family member is migrant worker (1 = yes)	0.16	0.20 **	0.15
	(0.11)	(0.10)	(0.12)
Family asset index in bottom 25% (1 = yes)	0.22 **	0.26 ***	0.22 **
	(0.09)	(0.09)	(0.10)
Student is female (1 = yes)	−0.09	−0.00	0.03
	(0.08)	(0.08)	(0.09)
Student is an only child (1 = yes)	−0.04	−0.12	−0.01
	(0.12)	(0.11)	(0.13)
Constant	−0.74 ***	−0.56 ***	−1.18 ***
	(0.13)	(0.12)	(0.14)
Observations	2926	2926	2926

Note: Values in parentheses represent the standard deviation of the estimated coefficient. * *p* < 0.10, ** *p* < 0.05, *** *p* < 0.01.

**Table 5:** OLS regression of caregivers’ mental health and student outcomes.

	Student Mental Health			Student Resilience Level (0–100)			Student Math Test Score (Standardized)		
Variable	Depression	Anxiety	Stress	Depression	Anxiety	Stress	Depression	Anxiety	Stress
Caregiver DASS depression symptoms (1 = yes)	0.24 ***			−1.39 *			−0.23 ***		
	(0.02)			(0.79)			(0.04)		
Caregiver DASS anxiety symptoms (1 = yes)		0.32 ***			−1.85 **			−0.21 ***	
		(0.02)			(0.74)			(0.04)	
Caregiver DASS stress symptoms (1 = yes)			0.16 ***			−0.68			−0.13 ***
			(0.02)			(0.86)			(0.04)
Student is female (1 = yes)	0.04 *	−0.02	0.01	−1.53 **	−1.49 **	−1.49 **	−0.09 **	−0.08 **	−0.08 **
	(0.02)	(0.02)	(0.02)	(0.72)	(0.72)	(0.72)	(0.04)	(0.04)	(0.04)
Student age (years)	−0.02 ***	−0.04 ***	−0.01	−0.09	−0.09	−0.11	−0.03	−0.03	−0.04
	(0.01)	(0.01)	(0.01)	(0.48)	(0.48)	(0.48)	(0.02)	(0.02)	(0.02)
Board at school (1 = yes)	0.10 ***	0.04	0.01	−1.90	−1.88	−1.92	−0.05	−0.05	−0.06
	(0.03)	(0.04)	(0.03)	(1.27)	(1.27)	(1.27)	(0.06)	(0.06)	(0.06)
Only child (1 = yes)	0.00	−0.01	−0.02	−0.85	−0.88	−0.81	−0.07	−0.07	−0.07
	(0.03)	(0.04)	(0.03)	(1.06)	(1.06)	(1.07)	(0.05)	(0.05)	(0.05)
Extracurricular reading time after school >30 min (1 = yes)	−0.03	−0.02	−0.05 **	2.31 ***	2.35 ***	2.36 ***	0.24 ***	0.24 ***	0.24 ***
	(0.02)	(0.02)	(0.02)	(0.73)	(0.73)	(0.73)	(0.04)	(0.04)	(0.04)
Screen time exceeds 1 h/day (1 = yes)	0.06 *	0.05	0.05 *	−2.46 **	−2.37**	−2.47 **	−0.06	−0.06	−0.06
	(0.03)	(0.04)	(0.03)	(1.10)	(1.10)	(1.11)	(0.06)	(0.06)	(0.06)
Parents are main caregivers (1 = yes)	−0.02	0.00	−0.01	1.53 *	1.46	1.60 *	0.03	0.03	0.04
	(0.03)	(0.03)	(0.02)	(0.89)	(0.89)	(0.89)	(0.04)	(0.04)	(0.04)
Father’s education level (1 = high school or above)	0.03	−0.02	0.03	0.63	0.67	0.70	0.09 *	0.09 **	0.10 **
	(0.03)	(0.03)	(0.02)	(0.94)	(0.94)	(0.94)	(0.05)	(0.05)	(0.05)
Mother’s education level (1 = high school or above)	−0.05	−0.03	−0.00	3.63 ***	3.57 ***	3.62 ***	0.11 **	0.11 **	0.12 **
	(0.03)	(0.04)	(0.03)	(1.14)	(1.14)	(1.14)	(0.06)	(0.06)	(0.06)
Family asset index at bottom 25% (1 = yes)	0.01	−0.02	0.02	−1.43 *	−1.39 *	−1.44 *	−0.08 *	−0.07 *	−0.08 *
	(0.02)	(0.03)	(0.02)	(0.83)	(0.83)	(0.83)	(0.04)	(0.04)	(0.04)
Constant	0.42 ***	0.83 ***	0.21 ***	57.34 ***	57.62 ***	57.30 ***	0.52 **	0.53 **	0.52 **
	(0.09)	(0.10)	(0.08)	(5.28)	(5.28)	(5.29)	(0.26)	(0.26)	(0.26)
Observations	1548	1548	1548	1548	1548	1548	2988	2988	2988
*R*-squared	0.087	0.119	0.041	0.083	0.085	0.081	0.103	0.103	0.095

Note: Values in parentheses represent the standard deviation of the estimated coefficient. * *p* < 0.10, ** *p* < 0.05, *** *p* < 0.01.

**Table 6:** Potential mechanisms for the effect of caregivers’ mental health on student outcomes.

	Expect Student go to College			Expect Student to Continue Schoolingafter Junior High			Student Seeks Help from Family		
Variable	Depression	Anxiety	Stress	Depression	Anxiety	Stress	Depression	Anxiety	Stress
Caregiver DASS moderate depression symptoms (1 = yes)	−0.11 ***			−0.05 ***			−0.04 **		
	(0.01)			(0.01)			(0.02)		
Caregiver DASS moderate anxiety symptoms (1 = yes)		−0.09 ***			−0.05 ***			−0.04 **	
		(0.01)			(0.01)			(0.02)	
Caregiver DASS moderate stress symptoms (1 = yes)			−0.10 ***			−0.03 **			−0.04 *
			(0.02)			(0.01)			(0.02)
Student is female (1 = yes)	0.03 **	0.04 ***	0.04 ***	0.01	0.01	0.01	0.02	0.02	0.02
	(0.01)	(0.01)	(0.01)	(0.01)	(0.01)	(0.01)	(0.02)	(0.02)	(0.02)
Student age (years)	−0.02 ***	−0.02 ***	−0.02 ***	−0.00	−0.00	−0.00	−0.05 ***	−0.05 ***	−0.05 ***
	(0.00)	(0.00)	(0.00)	(0.00)	(0.00)	(0.00)	(0.01)	(0.01)	(0.01)
Board at school (1 = yes)	−0.05 **	−0.05 ***	−0.06 ***	0.01	0.01	0.01	−0.00	−0.00	−0.00
	(0.02)	(0.02)	(0.02)	(0.02)	(0.02)	(0.02)	(0.03)	(0.03)	(0.03)
Only child (1 = yes)	0.00	0.00	0.00	−0.02	−0.02	−0.02	0.08 ***	0.08 ***	0.08 ***
	(0.02)	(0.02)	(0.02)	(0.01)	(0.01)	(0.01)	(0.03)	(0.03)	(0.03)
Extracurricular reading time after school >30 min (1 = yes)	0.04 ***	0.04 ***	0.04 ***	0.02	0.02	0.02 *	0.06 ***	0.06 ***	0.06 ***
	(0.01)	(0.01)	(0.01)	(0.01)	(0.01)	(0.01)	(0.02)	(0.02)	(0.02)
Screen time exceeds 1 h/day (1 = yes)	−0.03	−0.03	−0.03	0.01	0.01	0.01	−0.07 **	−0.07 **	−0.07 **
	(0.02)	(0.02)	(0.02)	(0.02)	(0.02)	(0.02)	(0.03)	(0.03)	(0.03)
Parents are main caregivers (1 = yes)	0.05 ***	0.05 ***	0.05 ***	0.03 **	0.03 **	0.03 ***	0.10 ***	0.10 ***	0.10 ***
	(0.02)	(0.02)	(0.02)	(0.01)	(0.01)	(0.01)	(0.02)	(0.02)	(0.02)
Father’s education level (1 = high school or above)	0.05 ***	0.05 ***	0.05 ***	−0.01	−0.01	−0.01	−0.00	0.00	0.00
	(0.02)	(0.02)	(0.02)	(0.01)	(0.01)	(0.01)	(0.02)	(0.02)	(0.02)
Mother’s education level (1 = high school or above)	0.03	0.03	0.03	−0.00	−0.00	0.00	0.03	0.03	0.03
	(0.02)	(0.02)	(0.02)	(0.02)	(0.02)	(0.02)	(0.03)	(0.03)	(0.03)
Family asset index at bottom 25% (1 = yes)	−0.06 ***	−0.06 ***	−0.06 ***	0.01	0.01	0.00	0.02	0.02	0.02
	(0.02)	(0.02)	(0.02)	(0.01)	(0.01)	(0.01)	(0.02)	(0.02)	(0.02)
Constant	1.01 ***	1.01 ***	1.00 ***	0.91 ***	0.91 ***	0.90 ***	0.79 ***	0.79 ***	0.78 ***
	(0.05)	(0.05)	(0.05)	(0.04)	(0.04)	(0.04)	(0.08)	(0.08)	(0.08)
Observations	2989	2989	2989	2989	2989	2989	2656	2656	2656
*R*-squared	0.065	0.063	0.060	0.013	0.013	0.007	0.043	0.043	0.043

Note: Values in parentheses represent the standard deviation of the estimated coefficient. * *p* < 0.10, ** *p* < 0.05, *** *p* < 0.01.

## Data Availability

Data are available upon request.
